# Application of methods of identifying receptor binding models and analysis of parameters

**DOI:** 10.1186/1742-4682-1-11

**Published:** 2004-11-16

**Authors:** Konstantin G Gurevich

**Affiliations:** 1 UNESCO Chair in healthy life for sustainable development, Moscow State Dentistry Medical University (MSDMU), Delegatskaya street 20/1, 127473, Moscow, Russian Federation, Russia

## Abstract

**Background:**

Possible methods for distinguishing receptor binding models and analysing their parameters are considered.

**Results and Discussion:**

The conjugate gradients method is shown to be optimal for solving problems of the kind considered. Convergence with experimental data is rapidly achieved with the appropriate model but not with alternative models.

**Conclusion:**

Lack of convergence using the conjugate gradients method can be taken to indicate inconsistency between the receptor binding model and the experimental data. Thus, the conjugate gradients method can be used to distinguish among receptor binding models.

## Background

Most medicinal preparations and biologically active substances do not penetrate into cells and must therefore exert their influence on intracellular processes by interaction with specific protein molecules at the cell surface [1-3], for which the name "receptors" is in common use. Hormones and drugs that interact with receptors are known as "ligands". Data from research in molecular biology, and also results from indirect studies, have established the following schemes of ligand-receptor interaction [see [4-6] represented by the general models:

*Non-cooperative interaction between ligand and receptor*:



where *R *is the receptor molecule, *L *is the ligand molecule, *RL *is the ligand-receptor complex, and *k*_+1 _and *k*_-1 _are respectively the kinetic constants of formation and dissociation of the complex.

Cooperative interaction between ligand and receptor



Interaction of one ligand with N types of binding sites



Let us note that the ligand-receptor interaction can also involve a combination of all three of these schemes. The most frequently used method for studying ligand-receptor interactions is the radioreceptor method [7], based on measuring the amount of radioactively labelled ligand bound in some defined manner to the appropriate receptor. Thus, experimentally, direct measurements of ligand-receptor complex concentration, [*RL*] are determined. The investigator has to solve two basic interrelated problems [6]:

1. discrimination among the ligand-receptor binding models (1–3 or modifications thereof);

2. determination of parameters that adequately relate the model to the experimental data.

From a pharmacological point of view, the most important parameters are the following:

[*R*_0_] (initial receptor concentration), and

*K*_*d *_= *k*_-1_/*k*_+1 _(dissociation constant) [7]

The concentration of receptors and the dissociation constant can be changed. Modification of these parameter values can occur in many physiological and pathophysiological situations. For instance, the receptor concentration can reflect functional receptor modifications, and the dissociation constant can reflect genetic alterations of the receptor [6].

To solve the two interrelated problems a series of graphic methods can be deployed, of which the most frequently used is the Scatchard method [7,8]. However, the application of graphic methods in many cases is limited because of experimental errors and/or receptor binding complexity [9,10]. In particular, graphic methods are inapplicable for definition of the cooperative binding parameters and for analysis of non-equilibrium binding.

Regression methods can be found for the measurement of ligand-receptor interaction constants [11]. As a matter of fact, these procedures computerize the graphic methods. Therefore, both regression methods and graphic methods are of limited applicability. The present paper argues that it is very difficult or impossible to discriminate reliably among receptor binding models or to analyse the parameters by traditional analytical methods.

## Materials and methods

Let us write the law of mass action for each ligand-receptor interaction scheme as:

*For the scheme *(1)



But [*R*] = [*R*_0_] - [*RL*], [*L*] = [*L*_0_] - [*RL*].

So equation (4) can be rewritten:



This differential equation relates to the class of *Rikkatty *equations. It can be solved analytically with the help of a special substitution [12], but in all other cases the substitutions [*R*] = [*R*_0_] - [*RL*], [*L*] = [*L*_0_] - [*RL*] do not generate analytically soluble equations. Therefore, all equations of this form were solved numerically using the Runge-Kutta method [13,14].

The differential equations are as follows:

*For scheme *(2):



*For scheme *(3):



Numerical solution of equations (5–7) was carried out to determine [*RL*]_*u*_. Random error assuming the normal distribution law was superimposed on the magnitude of [*RL*]_*u*_, and was calculated at 5, 10, 20 or 100 points.

The magnitude [*RT*]_*m *_was calculated using parameters other than [*RL*]_*u *_from models (1–3). These parameters were applied to the determination of [*RL*]_*u *_by the following functional minimization:

Φ = ([*RL*]_*u *_- [*RL*]_*m*_)^2^.     (8)

For functional minimization as per equation (8), Newton's method and its variants (the conjugate gradients method and coordinate descent method in various modifications) were used [15-17]. The iteration procedure stopped, when Φ/[*RL*]_*u *_was constant on the next iteration step.

It is clear from the literature [6] that [*R*_0_] and *K*_*d *_cannot be <10^-15 ^M or >10^-5 ^M. Hence the iteration procedure could be improved by re-scaling these parameters logarithmically, making 10^-15 ^M equivalent to -1 on the new scale and 10^-5 ^M equivalent to 1.

## Results and discussion

**Figure 1:**
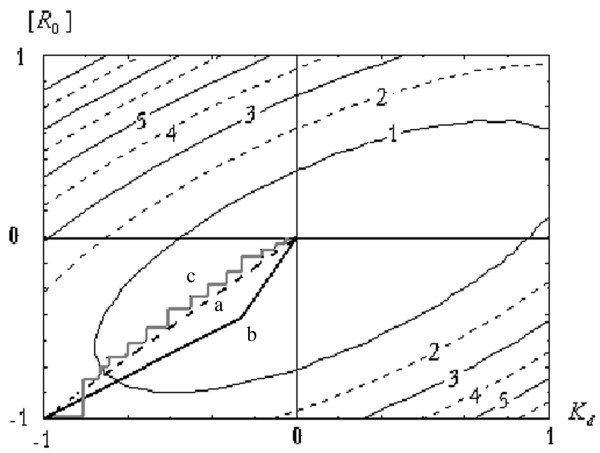
The functional (8) contour plot. The various methods of functional minimization are illustrated: a. The second derivative Newton method b. The conjugate gradients method c. The coordinate descent method

The functional (8) contour plots are shown in fig. 1. From this figure, the degree of correlation between the parameters [*R*_0_] *K*_*d *_can be seen. Therefore the magnification of the random error in evaluating the magnitude of [*RL*]_*u *_displaces the functional (8) global maximum from its true values. In a sufficiently large neighbourhood of the global maximum, the functional magnitude (8) is practically invariant. However, this modification becomes more essential for evaluating the ratio of the functional (8) to basis vector of values [*RL*]_*u*_. Therefore this ratio was used with the inhibiting criterion choice.

The Newton method converges only in the close neighbourhood of the global maximum. However, modifications of the Newton method using second derivatives allow convergence to the global maximum after 1–2 iterations (fig. 1, line 1).

The conjugate gradients method converged after 2–3 iterations (fig. 1, line 2). When magnification of the random error in the evaluation of [*RL*]_*u *_was taken into account, the convergence of the conjugate gradients method varied less than that of the Newton method.

The coordinate descent method required an indeterminately large number of iterations before satisfactory convergence was reached. Use of the exhausting coordinate descent method accelerated the convergence procedure, but the number of iterative steps remained large (fig. 1, line 3).

It can be shown that 5 points suffice to identify the parameters of model (1) using the conjugate gradients method, whereas this method required >10 points for identifying the parameters in a more complicated model. The Newton methods required >7 and 12 points respectively, and the coordinate descent method required >10 and 18 points.

**Figure 2:**
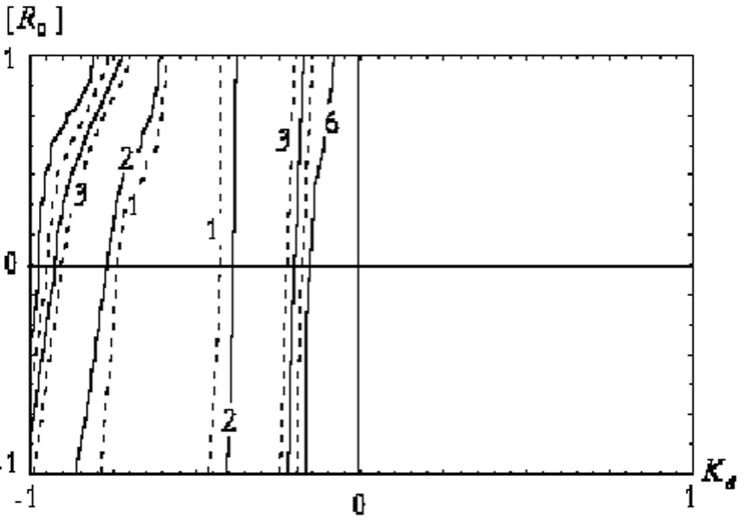
The functional (8) contour plot with an inadequate choice of receptor-binding model.

Functional (8) behaviour was analysed with respect to the evaluation of [*RL*]_*m *_using an incorrect binding model. In particular (see fig. 2), the functional (8) contour plot for model (1) with the attempt to approximate the given model by scheme (2). It follows from the figure that a discordant receptor binding model results in functional (8) contour plot modification.

Thus, the modification of the functional (8) contour plot from the type in fig. 1 to the type in fig. 2 can be used as the criterion for choosing a receptor binding model. With the right choice, the contour plot is similar to that represented in fig. 1. With the incorrect choice, the contour plot is similar to that shown in fig. 2.

It appears that when an incorrect choice of the receptor binding model has been made, the conjugate gradients method does not lead to convergence, whereas in some cases the Newton method converges to one of the local minima. Therefore, lack of convergence using the conjugate gradients method suggests an incorrect choice of receptor binding model.

## Conclusion

Possible methods have been explored for discriminating among models for receptor binding model and for defining the relevant parameters. The procedure devised allows one to determine the receptor binding model and its parameters, *even when the application of graphical methods is difficult or impossible*. As seen here, lack of convergence in the conjugate gradients method indicates that an incorrect choice of model has been made. It is also shown that for the defining the parameters of the correct model, 5–10 data points are sufficient.
